# VDJdb in 2019: database extension, new analysis infrastructure and a T-cell receptor motif compendium

**DOI:** 10.1093/nar/gkz874

**Published:** 2019-10-07

**Authors:** Dmitry V Bagaev, Renske M A Vroomans, Jerome Samir, Ulrik Stervbo, Cristina Rius, Garry Dolton, Alexander Greenshields-Watson, Meriem Attaf, Evgeny S Egorov, Ivan V Zvyagin, Nina Babel, David K Cole, Andrew J Godkin, Andrew K Sewell, Can Kesmir, Dmitriy M Chudakov, Fabio Luciani, Mikhail Shugay

**Affiliations:** 1 Pirogov Russian Medical State University, Moscow, Russia; 2 Shemyakin and Ovchinnikov Institute of Bioorganic Chemistry, Moscow, Russia; 3 Origins Center, Groningen, The Netherlands; 4 Institute for Advanced Study, University of Amsterdam, Amsterdam, The Netherlands; 5 Kirby Institute for Infection and Immunity, UNSW Sydney, Sydney, Australia; 6 School of Medical Sciences, UNSW Sydney, Sydney, Australia; 7 Center for Translational Medicine, Medical Department I, Marien Hospital Herne, University Hospital of the Ruhr-University Bochum, Herne, Germany; 8 Charité – Universitätsmedizin Berlin, Corporate Member of Freie Universität Berlin, Humboldt-Universität zu Berlin, and Berlin Institute of Health, Berlin-Brandenburg Center for Regenerative Therapies, Berlin, Germany; 9 Division of Infection and Immunity, School of Medicine, Cardiff University, Cardiff CF14 4XN, UK; 10 Immunocore Ltd., Abingdon, OX14 4RY, UK; 11 Theoretical Biology and Bioinformatics Department, Science Faculty, Utrecht University, Utrecht, Netherlands; 12 Center of Life Sciences, Skolkovo Institute of Science and Technology, Moscow, Russia

## Abstract

Here, we report an update of the VDJdb database with a substantial increase in the number of T-cell receptor (TCR) sequences and their cognate antigens. The update further provides a new database infrastructure featuring two additional analysis modes that facilitate database querying and real-world data analysis. The increased yield of TCR specificity identification methods and the overall increase in the number of studies in the field has allowed us to expand the database more than 5-fold. Furthermore, several new analysis methods are included. For example, batch annotation of TCR repertoire sequencing samples allows for annotating large datasets on-line. Using recently developed bioinformatic methods for TCR motif mining, we have built a reduced set of high-quality TCR motifs that can be used for both training TCR specificity predictors and matching against TCRs of interest. These additions enhance the versatility of the VDJdb in the task of exploring T-cell antigen specificities. The database is available at https://vdjdb.cdr3.net.

## INTRODUCTION

Knowing the exact antigen specificity of a given T-cell is key to solving numerous problems of both basic and applied immunology research: from discovering the specificity profile of TCR repertoire sequencing samples (1,2), to finding associations between autoimmunity and foreign mimics of self-antigens (3), and designing of personalized tumor immunotherapies (4). The field of molecular methods designed for studying antigen-specific T-cells is developing at a high pace: novel methodologies based on single-cell T-cell sequencing allow simultaneous detection of TCR sequence, T-cell phenotype and a vast array of antigen specificities (5). Resulting data, however, still need to be properly quality-controlled, and organized in the form of a database that is both comprehensive and easy to query.

After the first version of VDJdb (6) and a pathology-associated TCR database (McPAS-TCR (7)) were published, a commonly used iEDB database that describes immunogenic antigens was also modified to add metadata related to TCR and B-cell receptor sequences (8), highlighting the overall demand for such data in the field. A number of methods for TCR specificity prediction were also reported recently, many of which rely on VDJdb data for training and validating classifiers (9–12). The latter demonstrates the overall potential of the VDJdb database for developing better bioinformatic methods for TCR sequence analysis.

In this 2019 update, we focused on both accumulating the large amount of data generated by recent studies and providing an interface facilitating web-based analysis of adaptive immune receptor repertoire sequencing (AIRR-Seq, (13)) datasets. Given the large amount of data currently stored in VDJdb, we provided a reduced dataset of high-quality motifs that facilitates identification of TCR residues critical for recognition of certain antigens.

## MATERIALS AND METHODS

### Data acquisition and processing

All data acquired from published studies was manually parsed into VDJdb format according to VDJdb guidelines (https://github.com/antigenomics/vdjdb-db/blob/master/README.md) and quality-controlled both manually and using previously reported automated scripts (6). Summary statistics for VDJdb records were computed using an R notebook provided at https://github.com/antigenomics/vdjdb-db/blob/master/summary/vdjdb_summary.Rmd. Datasets from 10X genomics were downloaded from https://support.10xgenomics.com/single-cell-vdj/datasets (‘Application Note - A New Way of Exploring Immunity’ section, datasets ‘CD8+ T cells of Healthy Donor’ 1–4, available under the Creative Commons Attribution license) and processed using in-house scripts (available at https://bitbucket.org/kirbyvisp/10x-tcr/src/master/) that perform stringent filtering on the CDR3 length and composition, and tetramer read counts, yielding over 20 000 unique antigen-specific receptors. We’ve also performed several rounds of manual proofreading for the database and fixed a number of typos, mostly related to ambiguous segment naming (e.g. cases when V segments named according to Arden nomenclature were imported as IMGT segment names).

### Updated VDJdb web browser implementation details

Since 2017, we upgraded VDJdb web server to run on the latest Play framework (v2.7.2, https://www.playframework.com) with Akka HTTP server to improve the overall performance. We have also fully re-implemented frontend using Angular (https://angular.io) to provide a faster and more responsive interface. Importantly, we have implemented a fully documented REST API that can be used to query the database and can be found at https://vdjdb-web.readthedocs.io/en/latest/api.html. We also facilitated local VDJdb web server installation by providing a Docker image available at https://cloud.docker.com/u/bvdmitri/repository/docker/bvdmitri/vdjdb-web.

### TCR motif database

We have used the TCRNET implementation (14) in VDJtools (15) to identify TCR nodes in VDJdb TCR similarity network that have more neighbors than expected by chance, allowing for a single amino acid substitution in the CDR3 region. Only epitopes assigned to at least 30 distinct TCR amino acid sequences were considered. Selected nodes and their first neighbors were left in the TCR similarity network and sets of homologous TCR sequences (motifs) were defined for each epitope as connected components of the resulting graph. Position weight matrices (PWMs) for CDR3 amino acid sequences of inferred motifs were constructed using connected components of the graph. PWM normalization was performed by using the probability in a control set as the information measure, where control set is a set TCR sequences having the same V/J genes and CDR3 length coming from a pool of healthy donor samples. Details of this procedure are summarized in an R markdown notebook available at https://github.com/antigenomics/vdjdb-motifs.

## RESULTS

### Timeline of data accumulation and perspectives

**Figure 1.**
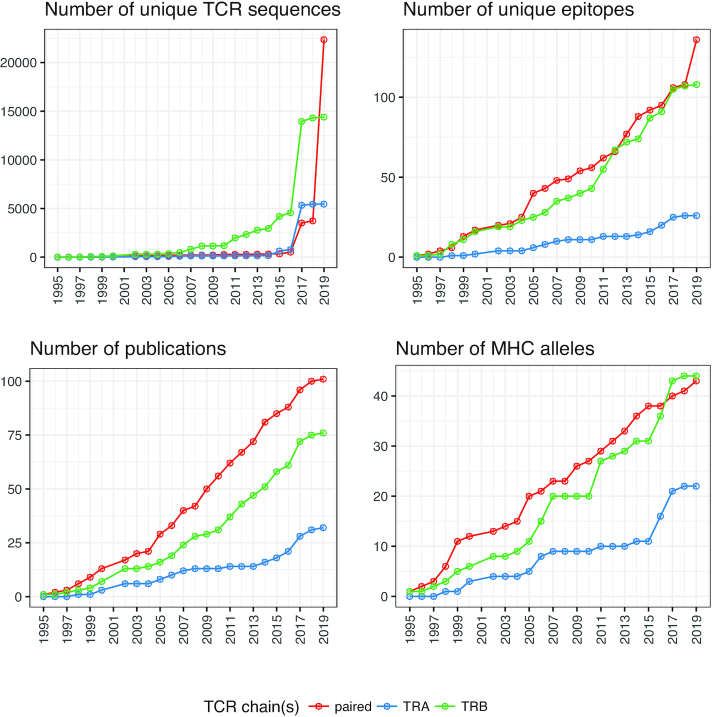
Summary statistics of VDJdb records according to July 2019 database release. Plots show the cumulative number of unique TCR sequences (up to unique V/J gene and CDR3 amino acid), antigens, MHC alleles and publications added to the database arranged by the publication date of corresponding papers. Colored lines represent records that only have TCR alpha (TRA, blue) or beta (TRB, green) chain or both chains (‘paired’ records, red). Note that as several VDJdb records can link a single TCR sequence to different metadata (e.g. another study and donor, or distinct epitope in case of cross-reactivity), the total number of unique TCR sequences (*n* = 42 211) is less than the total number of VDJdb records (*n* = 61 049).

VDJdb database is substantially expanded compared to the previous report (6): since the establishment of the database a total of 155 published studies were processed and added resulting in 61 049 TCR specificity records in 2019 compared to only 5491 in 2017. We used the publication dates of papers added to VDJdb to calculate the rate of accumulation of TCR specificity knowledge (Figure 1). The figure clearly shows that the number of records grows slowly prior to 2017, while a rapid growth in the number of records occurred in the last two years. The latter can be explained by the establishment of AIRR-Seq techniques producing very large amounts of TCR sequences (13) as a method of choice for performing the readout of TCR specificity assays.

Notably, while the number of unique epitopes with known specific TCRs increased, it is still dwarfed by the number of unique TCRs in the database, as there are currently only 212 epitopes in VDJdb. This highlights one of the most important factors currently limiting our ability to study and predict TCR specificity for a diverse range of antigens. We hope that in future this can be resolved using technologies allowing simultaneous testing for several antigen specificities (5).

Prior to 2019, there was a substantial lack of paired TCR records as most high-throughput studies focused on TCR beta chain only. However, with the advent of the droplet based single-cell sequencing techniques and the subsequent AIRR-seq focused improvements, the number of paired TCR alpha and beta records increased a lot. For example, one of the most recent studies added to VDJdb, a paired-chain dataset from 10X Genomics compendium (see Materials and Methods section), features 40 unique epitopes, more than any other high-throughput dataset produced so far.

### Online batch analysis of AIRR-Seq samples

Due to a growing number of immunological studies that choose AIRR-Seq technology to survey adaptive immunity, one of the main tasks for the current VDJdb update was to provide the ability to query a large TCR sequence set (e.g. whole blood or tissue specific T cell repertoire) against the entire database. We extended the functionality of the VDJdb browser and included batch upload and query options for AIRR-Seq datasets (Annotation tab of the web interface). Batch query supports several commonly used data formats (IMGT/HighV-QUEST, MiXCR, ImmunoSEQ, etc), borrowing functionality from the VDJviz browser (16).

**Figure 2.**
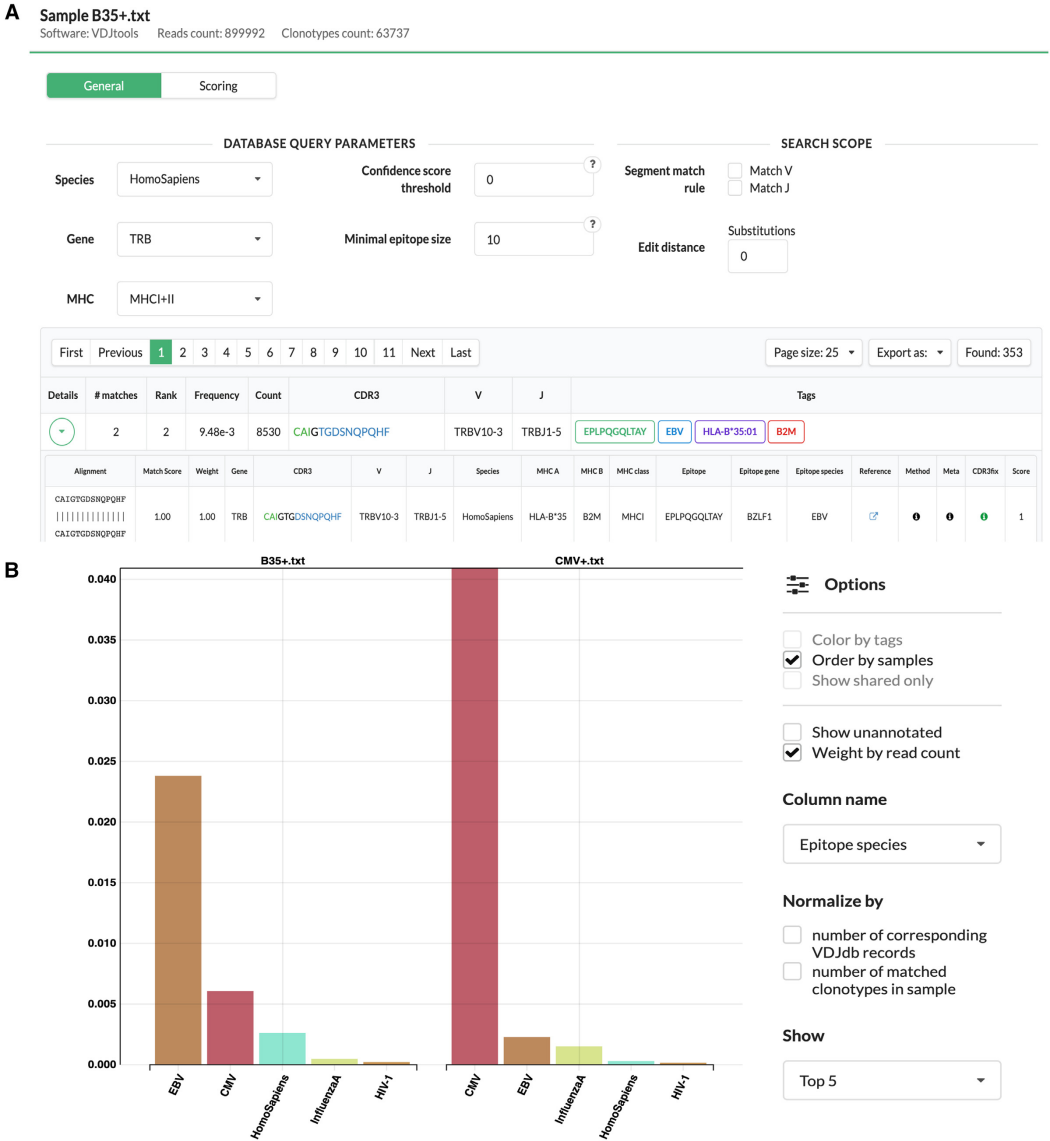
Batch query interface of the VDJdb web browser. (**A**) TCR sequence annotations provided for an example AIRR-Seq sample with default matching criteria. The sample (HIP02877) represents an individual carrying a HLA-B*35 allele and is taken from the Emerson *et al.*'s study (17). Note a prominent EBV-specific clonal expansion restricted to this allele at the top of the annotations list. (**B**) Summary statistics charts comparing HIP02877 (‘B35+.txt’, left) to HIP13994 (‘CMV+.txt’, right) sample representing a CMV+ individual from the same study.

Upon uploading a sample, TCR sequences are aligned against VDJdb records and users are provided with a list of VDJdb hits containing all necessary information regarding alignment and metadata (Figure 2A). Users can specify parameters of the TCR sequence homology search and/or pre-filter the VDJdb database to search in the specific data subset. Charts with summary statistics are supplied together with the analysis results and can be customized using different normalization options (Figure 2B). The results of repertoire annotation can be easily exported as tab-delimited tables and used for downstream analysis.

### Evaluating and exploring TCR motifs

Similarity analysis of TCR sequences specific for a certain antigen reveals a complex network structure containing both large interconnected hubs of highly similar TCRs and a diverse set of unique TCR variants that cannot be co-clustered (6). Reducing the complexity of the VDJdb TCR network can both greatly speed-up database queries for large AIRR-Seq datasets and make it easier to visualize and interpret TCR binding motifs. The latter can be achieved by applying a recently developed de novo TCR motif discovery algorithm that allows to distinguish hubs of similar TCRs that arise due to antigen-specific enrichment from hubs that arise simply due to biases in the V(D)J rearrangement process (14).

**Figure 3.**
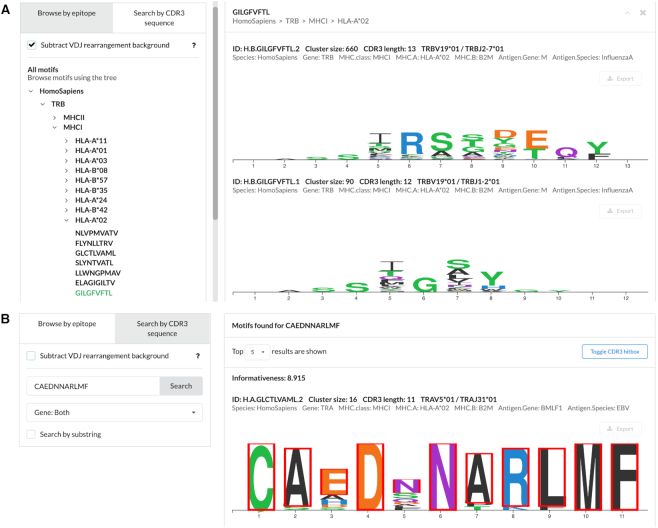
VDJdb motif browser interface. (**A**) Navigation tab showing the tree of available epitope motifs that can be selected to view PWMs of CDR3 amino acid sequences. The top plot shows the most abundant TCR beta chain motif for the A*02:GIL Influenza epitope. Motifs normalized for V(D)J rearrangement background are shown. (**B**) An example of CDR3 sequence query with matching amino acids highlighted. CDR3 sequence (CAEDNNARLMF) of the TCR alpha chain from the 3O4L PDB structure (TCR bound to A*02:GLC EBV epitope) was used as a query.

Upon selecting such hubs in the VDJdb TCR network with a stringent criterion that only allows for a single substitution in the CDR3 region (see Material and Methods section) we obtained a set of 501 motifs specific for 40 epitopes for human and mouse TCR alpha and beta chains. Additionally, we prepared a set of normalized motif PWMs that control for amino acid biases for a given V/J combination and CDR3 length. This normalization removes germline-encoded residues that are not unique to a given motif: the conserved Cys and Phe/Trp and other flanking CDR3 residues that rarely interact with an epitope. Users can navigate the TCR motifs using the VDJdb motif browser (Motifs tab of the web interface) as shown in Figure 3 and query both full-length or partial CDR3 sequences against the database. Note that we provide motif PWMs to illustrate epitope-specific TCR patterns and do not report their accuracy for TCR specificity classification as this is beyond the scope of the present work.

## DISCUSSION

During the last two years we have greatly increased the number of entries in the VDJdb, and made substantial improvements to the database structure and web interface. Currently, VDJdb represents the largest open data set of TCRs with characterized specificity. We believe that the comprehensiveness of the VDJdb make it an attractive database for the benchmarking of TCR specificity prediction algorithms and other basic studies investigating TCR:peptide:MHC interactions. The future development of the database will primarily follow two major directions. First, we aim at extending the set of existing records with putative antigen-specific TCRs using bioinformatic methods for *de novo* prediction of cognate TCR sequences. The latter is critical to provide a reasonable coverage for the overall diversity of an AIRR-Seq sample that can reach ∼10^6^ TCR variants. Second, we aim at modelling TCR:peptide:MHC structures based on existing templates and VDJdb records in order to provide a basis for investigating the complexity of interactions involving residues of the TCR heterodimer and bring more insight into TCR recognition of antigens. We believe that the recent expansion of the VDJdb database both in terms of the underlying dataset and functionality, will make it a favorable resource for tasks related to annotation of T-cell AIRR-seq samples and basic studies of TCR specificity.

## DATA AVAILABILITY

VDJdb database is available at https://vdjdb.cdr3.net.
